# Examining how and why polygenic dopamine composite levels moderate adolescents’ vulnerability to peer victimization

**DOI:** 10.1186/s13034-022-00521-7

**Published:** 2022-11-17

**Authors:** Yemiao Gao, Yuke Xiong, Xia Liu, Jinmeng Liu, Jinwen Li, Hui Wang

**Affiliations:** 1 Institute of Developmental Psychology, Faculty of Psychology, Beijing Normal University, No. 19 Xinjiekouwai Street, Beijing, China; 2 Collaborative Innovation Center of Assessment Toward Basic Education Quality, Beijing Normal University, Beijing, China

**Keywords:** Peer victimization, Emotion dysregulation, Dopamine genes, Externalizing problems

## Abstract

Extensive literature documents that dopaminergic genes play an important role in the link between adverse environments and adolescents’ problem behavior. However, little is known about the potential mechanism underlying adolescents’ vulnerability to peer victimization. The current study examined the effect of the interplay between a polygenic dopamine composite (i.e., COMT Val158Met and DRD2-141C Ins/Del polymorphisms) and peer victimization on adolescents’ externalizing problems as well as the mediating role of emotion dysregulation in the interactive effects in a sample of 393 Chinese adolescents (Mean age = 14.71 years; 50.1% girls). A significant moderation of dopaminergic genetic composite was observed in girls but not in boys. In addition, emotion dysregulation partially explained the moderating effect of dopaminergic genes. Specifically, girls with genic composite indexing low dopamine activity reported a higher level of emotion dysregulation when faced with more peer victimization. More difficulties with emotion regulation, in turn, predicted more pronounced externalizing problems in girls. This study underscores polygenic underpinnings of adolescent vulnerability to negative peer experiences and suggests the importance of considering sex differences when investigating genic influence on the relationship between adverse environments and externalizing problems.

**Supplementary Information:**

The online version contains supplementary material available at 10.1186/s13034-022-00521-7.

## Introduction

Peer victimization is a pervasive public health concern, with up to one out of three adolescents globally reporting experiences of being victimized by their peers [1]. Peer victimization refers to the receipt of intentional, repetitive (or likely repetitive) negative actions from one or more peers [2]. The forms of peer victimization may be physical (e.g., hitting), verbal (e.g., teasing), or relational (e.g., social exclusion) in nature. Although many studies have demonstrated that peer victimization is associated with adolescents’ externalizing problems such as delinquency (see [3] for a review), there is variability in the magnitude of such relations. Moreover, despite progress made in identifying individual attributes (e.g., academic performance and skin conductance level reactivity) that account for the heterogeneity in adolescents’ vulnerability to externalizing symptoms [4, 5], the molecular genetic sources of variability in associations between peer victimization and adolescents’ externalizing problems are not well understood. Thus, the primary aim of this study was to test whether a set of genes moderates the development of externalizing problems in victimized adolescents. Guided by the established roles of dopaminergic genes in regulating emotional reactivity and motivational dispositions to environmental cues of adversity [6], this study specifically focused on these genes in the context of victimization.

To better understand how and why dopaminergic genes function as moderators in the context of peer victimization, the second aim of this study was to explore whether emotion dysregulation accounts for the moderating role of dopaminergic genes in adolescents’ vulnerability to externalizing problems. From a multiple-level analysis perspective, the polyvagal theory of psychopathology proposes that emotion dysregulation amplifies genetic vulnerabilities, resulting in the development of externalizing problems [7, 8]. Therefore, this study specifically examined the hypothesis that emotion dysregulation is a mediating mechanism accounting for why the association between peer victimization and externalizing problems varies depending on the dopamine genotypes involved. Since boys and girls differ in their responses to victimization, especially during adolescence [9], and in their developmental pathways to externalizing problems [8], as our third aim, this study tested for potential sex differences in the patterns of connections.

### Dopamine genes as moderators of peer victimization

**Fig. 1:**
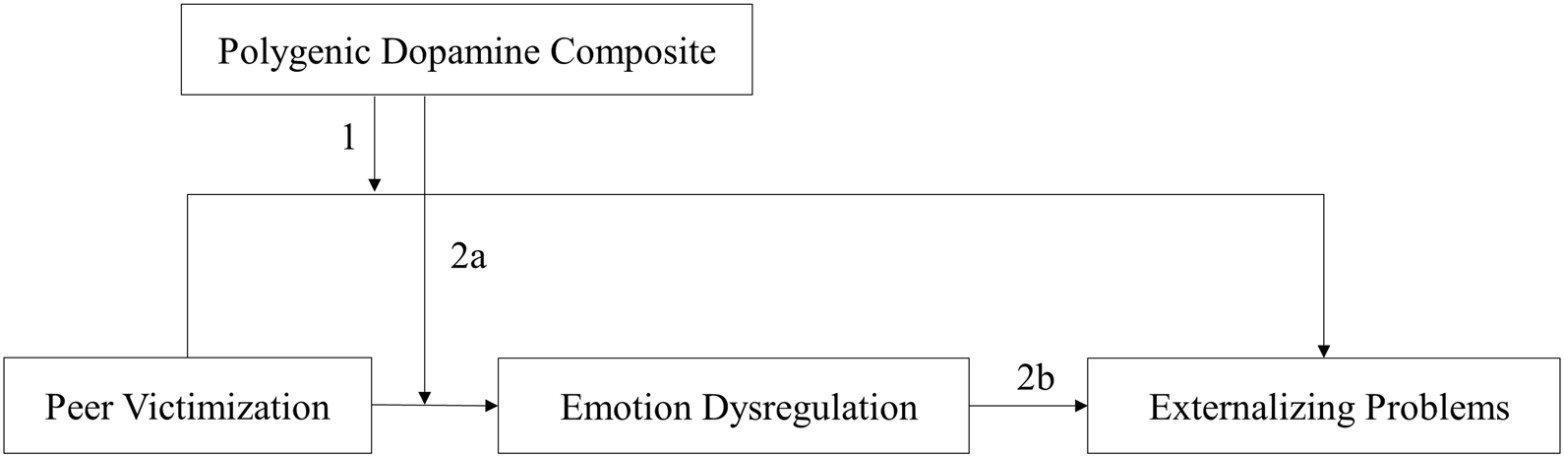
Conceptualization of adolescents’ emotion dysregulation as a mediator of the interaction between peer victimization and polygenic dopamine composite in predicting externalizing problems

As illustrated by path 1 of the conceptual model represented in Fig. 1, the first goal of this study was to test the hypothesis that the relationship between peer victimization and adolescents’ externalizing problems would be stronger for those carrying low dopamine activity alleles. Research on the etiology of externalizing problems has provided some insights into the importance of gene × environment (G × E) interactions [7, 10]. For instance, genetic markers that are related to low dopamine levels might be important genetic markers for individual differences in vulnerability to externalizing problems [10]. Mesolimbic dopamine plays a critical role in the regulation of neural circuits implicated in motor, cognitive, and affective behavioral processes, including emotional-motivational processes (e.g., reactivity to rewarding and emotional stimuli) [6, 11]. Individual differences in dopamine transmission are associated with individual variation in environmental sensitivity and vulnerability to psychopathologies [12]. Using one candidate gene (i.e., DRD4), researchers have found that dopamine-related genes interact with peer victimization to affect children’s externalizing behaviors [13].

Regarding polymorphisms related to dopamine transport and uptake, two widely researched genes in the context of externalizing problems are the dopamine D2 receptor (DRD2) and the catechol-O-methyl transferase (COMT) gene. First, the DRD2 gene regulates the expression of the D2 receptor in the mesocorticolimbic dopaminergic pathways [14]. The deletion (Del) allele of a promoter polymorphism (-141C Ins/Del, rs1799732) of the DRD2 gene is associated with reduced expression of D2 receptors and decreased striatal D2 binding [15], which lead to a lower-functioning dopaminergic system in the ventral striatum and prefrontal cortex (PFC) [16]. The reduced dopaminergic functioning translates to impairments of self-control and more sensation-seeking, which may increase people’s impulsive, rewarding behavioral tendencies [16, 17]. Second, the COMT gene regulates the production of the COMT enzyme, which is of critical importance to the elimination of dopamine, epinephrine, and noradrenaline primarily in the mesocortical pathway. It is well established that variation in COMT is important for emotional reactivity, especially for that related to negative emotions [11]. The most commonly studied polymorphism in the COMT gene is Val158Met. In comparison to the Met allele of the COMT gene, the Val allele is associated with higher levels of COMT enzyme activity [18] and lower prefrontal dopamine signaling [19], resulting in impaired PFC function and relative cognitive processes [20].

According to several theoretical frameworks, the DRD2 and COMT genes are critical factors accounting for individual differences in their reactivity to adverse environments (e.g., [10, 11]). Consistent with these theories, research has indicated that the DRD2 gene could regulate an individual’s resilience to victimization [21], with the hypodopaminergic alleles of the DRD2 gene increasing the links between negative environmental cues and adolescent delinquency (e.g., [22, 23]). Regarding the COMT gene, studies have shown that the Val allele confers vulnerability to externalizing problems [24] and internalizing problems [25, 26] in adverse environments. Nonetheless, other studies have identified the hyperdopaminergic alleles as a more sensitive factor for negative environmental cues (e.g., [27]). Findings from a meta-analysis of the COMT gene are also mixed regarding which allele amplifies or weakens the associations between environmental adversities and externalizing problems [28]. The discrepancy suggests that the results of studies on single candidate genes could be erratic. A polygenic score, however, could represent a stronger relationship to the dopaminergic pathway. Further examination of polygenic effects of genetic pathways could reach more robust and consistent conclusions. Additionally, most G × E studies have focused on family environments (e.g., parenting and maltreatment) and other stressful events, and few studies have used the polygenic approach to investigate adolescents’ vulnerability to the effects of peer victimization, which is a common occurrence in this developmental period. Thus, the current study aimed to address this gap by examining the moderating effect of dopaminergic genes on adolescents’ sensitivity to peer victimization.

### Emotional dysregulation as a mediating mechanism

Delineating the moderating role of dopamine genes is only the first step to understanding adolescents’ heterogeneity in negative outcomes of peer victimization. To better explain the underlying mechanism of G × E interaction effects, the next step of this study was to investigate how and why dopamine genes moderated adolescents’ susceptibility to externalizing problems in the context of peer victimization. As denoted by Paths 2a and 2b in Fig. 1, this study aimed to examine whether emotional dysregulation mediated the interaction between peer victimization and dopamine genes in predicting externalizing problem behaviors.

According to the developing transdiagnostic models of psychopathology [29], intrapersonal risk factors play a critical mediating role in the links between distal risk factors (e.g., environmental risk factors) and psychopathology. One category of proximal transdiagnostic risk factors is individual differences in particular styles of responding to environments, such as emotion regulation. Another model also stresses the importance of emotion processes to the relationship between chronic stress and adolescent conduct problems [30]. Emotion regulation involves a number of processes, including emotion monitoring, emotion evaluation, and emotion modification, to accomplish one’s goals [31]. Emotion regulation deficits (i.e., emotion dysregulation) can be regarded as a failure to identify one’s emotions and an absence of capacities to select or implement adaptive regulation strategies [32]. Prior work has identified exposure to adverse peer experiences as an important contributing factor to emotion dysregulation during adolescence [33]. Peer victimization can impair adolescents’ emotion regulation in multiple respects, such as emotional reactivity, cognitive regulation, and behavioral regulation [34]. Consistent evidence suggests that peer victimization is associated simultaneously [35] and prospectively [36] with adolescent emotion dysregulation. In addition, studies have documented disruptions in emotion regulation as a mechanism linking adverse family and peer contexts to an increase in a broad range of psychopathological symptoms, including aggressive behaviors [36] and externalizing problems [37].

In support of this hypothesis, the neurobiological model of environmental reactivity proposes that dopamine variation is important for reactivity to negative emotional environmental cues [11]. A decreased level of dopamine could result in more seeking of immediate rewards, higher levels of irritability, and more negative affect [6]. Thus, adolescents with low dopaminergic activity could be more reflexively reactive to aversive environmental factors and may exhibit higher levels of emotion dysregulation following exposure to peer victimization and develop more problematic behaviors.

### The present study

In summary, prior research on G × E rarely considers dopaminergic genetic effects on adolescents’ reactivity to negative peer experiences, and little is known about the potential pathways linking both dopamine genes and peer victimization to adolescent externalizing problems. To address these gaps, this study aimed to examine whether a polygenic composite consisting of two dopaminergic genes moderates the relationship between peer victimization and adolescents’ externalizing problems. Based on the above literature review, adolescents carrying more hypodopaminergic alleles were expected to show stronger associations involving peer victimization. Moreover, it was hypothesized that emotion dysregulation (partially) mediates the association between peer victimization in predicting externalizing problems and that adolescents carrying hypodopaminergic alleles show a stronger association between peer victimization and emotion dysregulation. Since adolescent boys and girls differ in their responses to victimization [38] and their developmental pathways to externalizing problems [8], and the interactive effects of genes and the environment may also differ by sex [39], as our third aim, this study tested for potential sex differences in the patterns of connections.

## Methods

### Participants

Four hundred and eighty-three adolescents (49.8% girls, *M*age = 14.69 years, *SD* = 0.86 years, range = 13–18 years) from four public junior high schools in southwest China participated in our study. All participants were in Grade 9. Of the adolescents, 7 (0.1%) were not successfully genotyped for COMT Val158Met or DRD2–141C ins/del polymorphisms, 3 (0.01%) did not report their sex, and 80 (16.6%) had incomplete questionnaire data. The final sample comprised 393 Chinese adolescents (50.1% girls, *M*age = 14.71 years, *SD* = 0.86 years). Results of chi-square tests and *t*-tests revealed that there were no significant differences (*p*s > 0.05) in the main variables of interest between the 90 cases dropped from the study and the final sample.

### Procedures

This study was approved by the Research Ethics Committee of the authors’ university. With the help of public welfare organizations and the local education center, several public junior high schools were randomly selected, and four agreed to cooperate in the research. The study used cluster random sampling to choose 14 classes in Grade 9 of the four schools. Within these classes, all students (*N* = 570) were invited to participated in the study, with 84.7% (*N* = 483) providing personal and parental consent and participating. During a 40-min class session, participants completed a self-report questionnaire assessing peer victimization, emotion dysregulation, and externalizing problems. Saliva samples were obtained for DNA extraction. The whole process was completed under the guidance of trained experimenters. After completing the survey, each participant received a gift consisting of stationery.

### Measures

#### Peer victimization

Peer victimization was assessed by a self-report measure developed by [40] and revised by [41]. The 18-item questionnaire measures how often (1 = never, 2 = rarely, 3 = sometimes, 4 = often) children have experienced physical (e.g., “other students have beat me up this semester”), verbal (e.g., “other students deliberately speak ill of me and spread rumors about me”), relational (e.g., “other students intentionally do things to make the teacher dislike me”), and victimizing experiences and attacks on their property (e.g., “other students have stolen money or something else from me”) over the past semester. The mean of the 18 items was taken, with higher scores indicating more peer victimization. For the present sample, the Cronbach’s alpha was 0.92. Confirmatory Factor Analysis (CFA) exhibited good fit for a second-order model with four established factors (*χ*^2^/*df* = 3.23, CFI = 0.90, TLI = 0.89, RMSEA = 0.08, and SRMR = 0.05).

#### Emotion dysregulation

Adolescents’ emotion dysregulation was assessed with the difficulties in emotion regulation scale (DERS, [42]). The scale measures six specific dimensions: (a) nonacceptance of emotional responses (e.g., “When I’m upset, I feel guilty for feeling that way”), (b) difficulties engaging in goal-directed behavior (e.g., “When I’m upset, I have difficulty concentrating”), (c) impulse control difficulties (e.g., “When I’m upset, I lose control over my behaviors”), (d) lack of emotional awareness (e.g., “I am attentive to my feelings”-reverse coded), I limited access to emotion regulation strategies (e.g., “When I’m upset, I believe that I’ll end up feeling very depressed”), and (f) lack of emotional clarity (e.g., “I have difficulty making sense of my feelings”). Participants were asked to rate how often each of the 36 items applied to them on a 5-point Likert scale ranging from 1 (*almost never*) to 5 (*almost always*); mean scores were used in the analyses, with higher scores representing higher levels of emotion dysregulation. The Cronbach’s alpha was 0.88 for the present sample. CFA showed that the scale fitted the datum well (*χ*^2^/*df* = 2.78, CFI = 0.82, TLI = 0.80, RMSEA = 0.07, and SRMR = 0.10).

#### Externalizing problems

Externalizing problems were measured by 12 items from the delinquent subscale of the child behavior checklist-youth self-report measure [43] and adapted problem behaviors scale [44]. The questionnaire covered common externalizing problems among adolescents (e.g., stealing and violating school rules) and has been successfully used among Chinese adolescents [45]. Adolescents responded to each item using a scale of 1 (*never*) to 4 (*always*). Mean scores were used, with higher scores indicating more pronounced externalizing problems. The Cronbach’s alpha was 0.85.

#### Covariate: subjective socioeconomic status (SES)

SES was included as a covariate given its association with externalizing problems [46]. The MacArthur Scale was adopted to assess participants’ subjective SES [47]. Participants were presented with a diagram of a “social ladder” with 10 rungs and were asked to rank themselves by choosing the rung that represented their relative position in society. The 10 rungs of the social ladder were rated from 1 to 10 from the bottom. Higher scores indicate higher subjective SES.

#### Genotyping

Genomic DNA was extracted from each saliva sample using standard techniques. The DRD2–141C ins/del and COMT Val158Met polymorphisms were genotyped by the analysis of primer extension products generated from amplified genomic DNA using a Sequenom (San Diego, CA, USA) chip-based MALDI-TOF mass spectrometry platform. The–141C ins/del polymorphism was amplified using the following primer sequences: forward ACGTTGGATGCTCAAAACAAGGGATGGCGG and reverse ACGTTGGATGAAAGGAGCTGTACCTCCTCG. The primer sequences for the Val158Met polymorphism were as follows: forward ACGTTGGATGACCCAGCGGATGGTGGATTT and reverse ACGTTGGATGTTTTCCAGGTCTGACAACGG. Guided by research indicating that the Del allele of DRD2 is associated with decreased expression of DRD2 [15], allelic variation in DRD2–141C Ins/Del was coded based on (2) Del/Del, (1) Del/Ins, and (0) Ins/Ins. For the COMT Val158Met gene, previous research has shown that the number of Val alleles is associated with hypodopaminergic tone [19]. Accordingly, the COMT genotype was coded as (2) Val/Val, (1) Val/Met, and (0) Met/Met (see Table 1). Genotype distributions for DRD2 (1.3% Del/Del, 22.6% Del/Ins, 76.1% Ins/Ins) and COMT (53.7% Val/Val, 40.7% Val/Met, 5.6% Met/Met) were in Hardy–Weinberg equilibrium (DRD2: *χ*^2^ = 0.32, *p* = 0.57; COMT: *χ*^2^ = 1.38, *p* = 0.24). The polygenic dopamine composite was computed by summing the scores of both dopamine genes, which resulted in a continuous variable ranging from 0 to 4. The higher the sum score, the lower the dopamine activity.

**Table 1:** Means and standard deviations of the primary variables in the study

Variable		Girls (*n* = 197)		Boys (*n* = 196)		*t* test
		*M/*%	*SD/N*	*M/*%	*SD/N*	
Age		14.72	0.84	14.67	0.90	− 0.54
SES		4.57	1.21	4.61	1.55	0.28
Peer victimization		1.60	0.48	1.63	0.47	0.65
Polygenic dopamine composite		1.76	0.81	1.70	0.79	− 0.71
Emotion dysregulation		2.63	0.52	2.53	0.45	− 2.10^a^
Externalizing problems		1.34	0.32	1.51	0.40	4.84^b^
COMT Val158Met	Val/Val (2)	52.3	103	55.1	108	–
	Val/Met (1)	42.1	83	39.3	77	–
	Met/Met (0)	5.6	11	5.6	11	–
DRD2 -141C Ins/Del	Del/Del (2)	2.0	4	0.5	1	–
	Del/Ins (1)	25.4	50	19.9	39	–
	Ins/Ins (0)	72.6	143	79.6	156	–

^a^*p* < 0.05

^b^*p* < 0.001

### Analytic plan

Because the aim was to test for sex differences, separate analyses were conducted on samples of boys and girls. First, descriptive statistics of all variables were computed, and independent *t* tests were conducted to examine sex differences. Additionally, Pearson correlations of the included variables were calculated. Second, following the procedure proposed by Wen and Ye [48] for testing mediated moderation models, we conducted three equation models in the PROCESS macro [49] for SPSS: (1) Regress externalizing problems on peer victimization (PV), polygenic dopamine composite levels (PDC), and PV × PDC. If there were any significant two-way interactions, (2) regress emotional dysregulation on PV, PDC, and PV × PDC, and (3) regress externalizing problems on PV, PDC, emotional dysregulation, and PV × PDC. Model 1 in the PROCESS macro was used to test the first equation, and the Model 8 in the PROCESS macro was applied to examine the second and the third equations. Age and subjective SES were included as control variables in all analyses considering their associations with externalizing problems. All models were applied with a 95% confidence interval and 5000 bias-corrected bootstrap samples.

## Results

Means and standard deviations of the primary variables are presented in Table 1 for girls and boys separately. Emotion dysregulation and externalizing problems showed significant gender differences. Compared to boys, girls had lower scores for externalizing problems and higher scores for emotion dysregulation.

Table 2 contains the correlations between the variables involved in the present study, with results shown above the diagonal for boys and below the diagonal for girls. The results of the bivariate correlations show that peer victimization was positively correlated with emotion dysregulation (*rs* = 0.43–0.44, *p* < 0.05) and externalizing problems (*rs* = 0.33–0.45, *p* < 0.05), and emotion dysregulation was positively correlated with externalizing problems (*rs* = 0.29–0.46, *p* < 0.05). In addition, the polygenic dopamine composite was unrelated to peer victimization (*rs* = 0.01–0.08, *p* > 0.05), excluding the existence of gene–environment correlations.

**Table 2:** Correlations of the primary variables in the study for adolescent girls and boys

Variable	1	2	3	4	5	6
1 Age	–	− 0.00	0.02	− 0.16^a^	0.09	0.01
2 SES	− 0.02	–	0.01	0.10	− 0.08	− 0.21^b^
3 Peer victimization	0.10	0.03	–	0.08	0.43^c^	0.33^c^
4 Polygenic dopamine composite	0.08	− 0.07	0.01	–	− 0.02	0.09
5 Emotion dysregulation	0.07	0.01	0.44^c^	0.12	–	0.29^c^
6 Externalizing problems	0.09	0.04	0.45^c^	0.08	0.46^c^	–

Boys’ correlations appear above the diagonal and girls’ correlations below the diagonal

^a^*p* < 0.05

^b^*p* < 0.01

^c^*p* < 0.001

### Primary analysis I: does the polygenic dopamine composite moderate the association between peer victimization and externalizing problems?

**Fig. 2:**
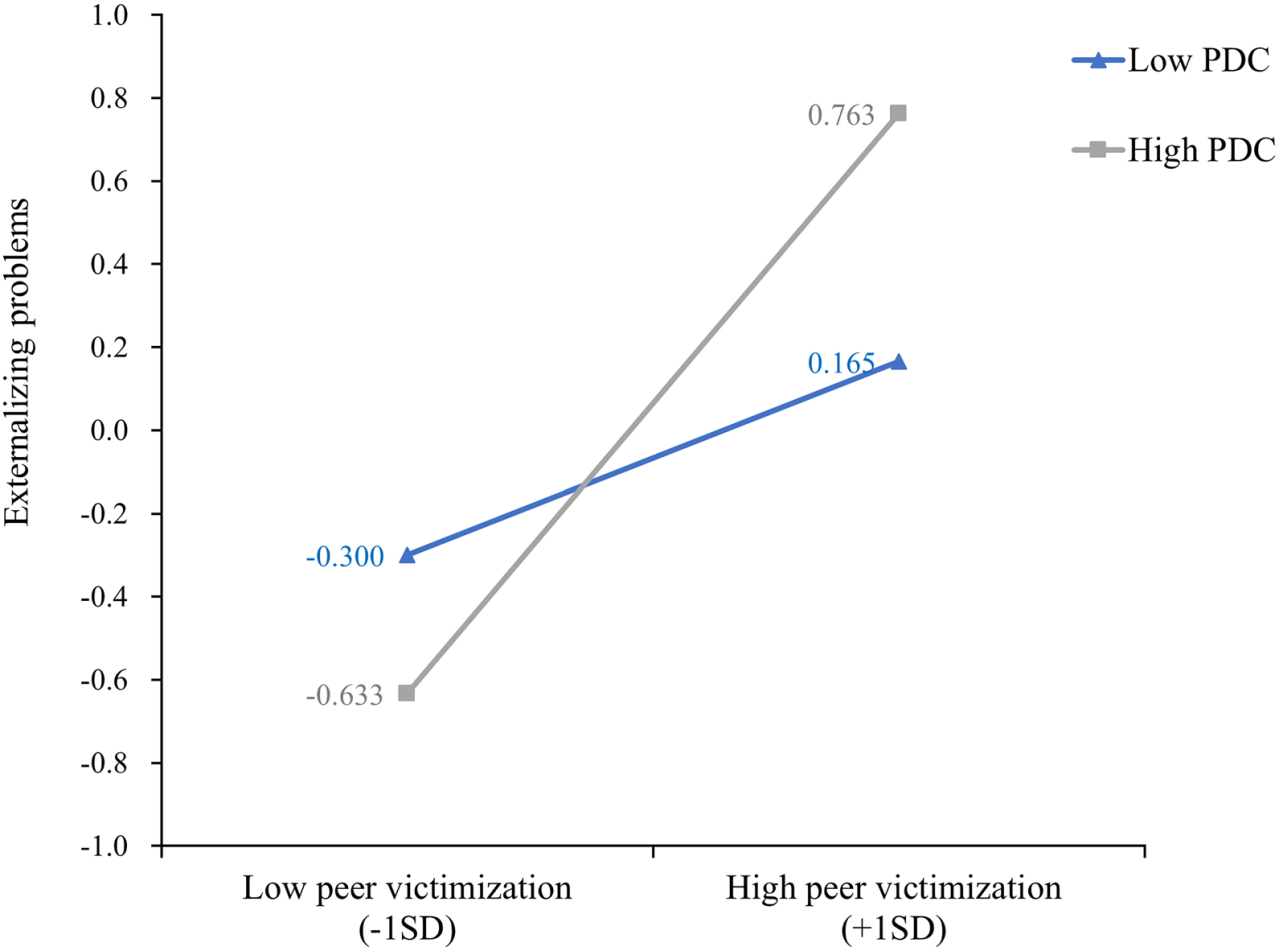
Interaction of peer victimization and polygenic dopamine composite on adolescent girls’ externalizing problems

First, whether the interplay of peer victimization and polygenic dopamine composite levels predicted adolescents’ externalizing problems was examined. Table 3 presents the regression models for girls and boys separately. The results showed a main effect of peer victimization on adolescent girls’ and boys’ externalizing problems (*β* = 0.47, *p* < 0.001; *β* = 0.33, *p* < 0.001) but a nonsignificant effect of the polygenic dopamine composite for girls and boys (*β* = 0.07, *p* > 0.05; *β* = 0.09, *p* > 0.05). Furthermore, there was a significant peer victimization × polygenic dopamine composite interaction effect on externalizing problems only among adolescent girls (*β* = 0.23, *p* < 0.01). To facilitate description, girls’ externalizing problems were plotted against peer victimization separately for low and high levels of polygenic dopamine composite (1 *SD* below the mean and 1 *SD* above the mean, respectively; see Fig. 2). A simple slope test showed that for girls with high levels of polygenic dopamine composite, peer victimization was more strongly associated with externalizing problems (*b*_*simple slope*_ = 0.70, *p* < 0.001) than for girls with low levels of polygenic dopamine composite (*b*_*simple slope*_ = 0.23, *p* < 0.01).

**Table 3:** Testing the moderation model in adolescent girls and boys

Variable	Equation 1 (Externalizing problems)					
	Girls (*n* = 197)			Boys (*n* = 196)		
	β	SE	95% CI	β	SE	95% CI
PV	0.47^b^	0.06	[0.34, 0.59]	0.33^b^	0.07	[0.20, 0.46]
PDC	0.07	0.06	[− 0.06, 0.19]	0.09	0.07	[− 0.05, 0.22]
PV × PDC	0.23^a^	0.07	[0.10, 0.36]	0.02	0.07	[− 0.12, 0.16]
SES	0.06	0.06	[− 0.07, 0.18]	− 0.22^a^	0.07	[− 0.35, − 0.09]
*R*^*2*^	0.26			0.16		
*F*	16.50^b^			9.26^b^		

PV and PDC represent peer victimization and polygenic dopamine composite respectively

^a^*p* < 0.01

^b^*p* < 0.001

### Primary analysis II: does emotional dysregulation mediate the moderating effect of polygenic dopamine composite levels?

Given the significant moderation findings of our first primary analysis of adolescent girls, the second analytic step was designed to address the question of why the magnitude of peer victimization as a predictor of girls’ externalizing problems varied as a function of their polygenic dopamine composite levels. Thus, a mediated moderation model was estimated to examine whether girls’ emotion dysregulation mediated the relationship between the interactive effects of peer victimization and polygenic dopamine composite levels on externalizing problems.

As displayed in Table 4, the results from Eq. 2 show that peer victimization was positively associated with girls’ emotion dysregulation (*β* = 0.45, *p* < 0.001), whereas the polygenic dopamine composite was not associated with girls’ emotion dysregulation (*β* = 0.11, *p* > 0.05). Furthermore, the peer victimization × polygenic dopamine composite interaction term predicted girls’ emotion dysregulation (*β* = 0.15, *p* < 0.05). Equation 3 in Table 4 shows that emotion dysregulation was positively associated with girls’ externalizing problems (*β* = 0.29, *p* < 0.001), and the interaction between peer victimization and polygenic dopamine composite levels on girls’ externalizing problems remained significant (*β* = 0.19, *p* < 0.01). The above results show that the interaction between peer victimization and polygenic dopamine composite levels partially indirectly affects girls’ externalizing problems through emotion dysregulation as a mediating variable.

**Table 4:** Testing the mediated moderation model in adolescent girls

Variable	Equation 2 (Emotional dysregulation)			Equation 3 (Externalizing problems)		
	β	SE	95% CI	β	SE	95% CI
PV	0.45^c^	0.06	[0.32,0.57]	0.34^c^	0.07	[0.20, 0.47]
PDC	0.11	0.06	[− 0.02,0.23]	0.03	0.06	[− 0.08, 0.15]
PV × PDC	0.15^a^	0.07	[0.02,0.28]	0.19^b^	0.06	[0.06, 0.32]
ED				0.29^c^	0.07	[0.16, 0.42]
SES	0.03	0.06	[− 0.10,0.15]	0.05	0.06	[− 0.07, 0.17]
*R*^*2*^	0.22			0.32		
*F*	13.73^c^			18.05^c^		

PV, PDC, and ED represent peer victimization, polygenic dopamine composite, and emotion dysregulation respectively

^a^*p* < 0.05

^b^*p* < 0.01

^c^*p* < 0.001

**Fig. 3:**
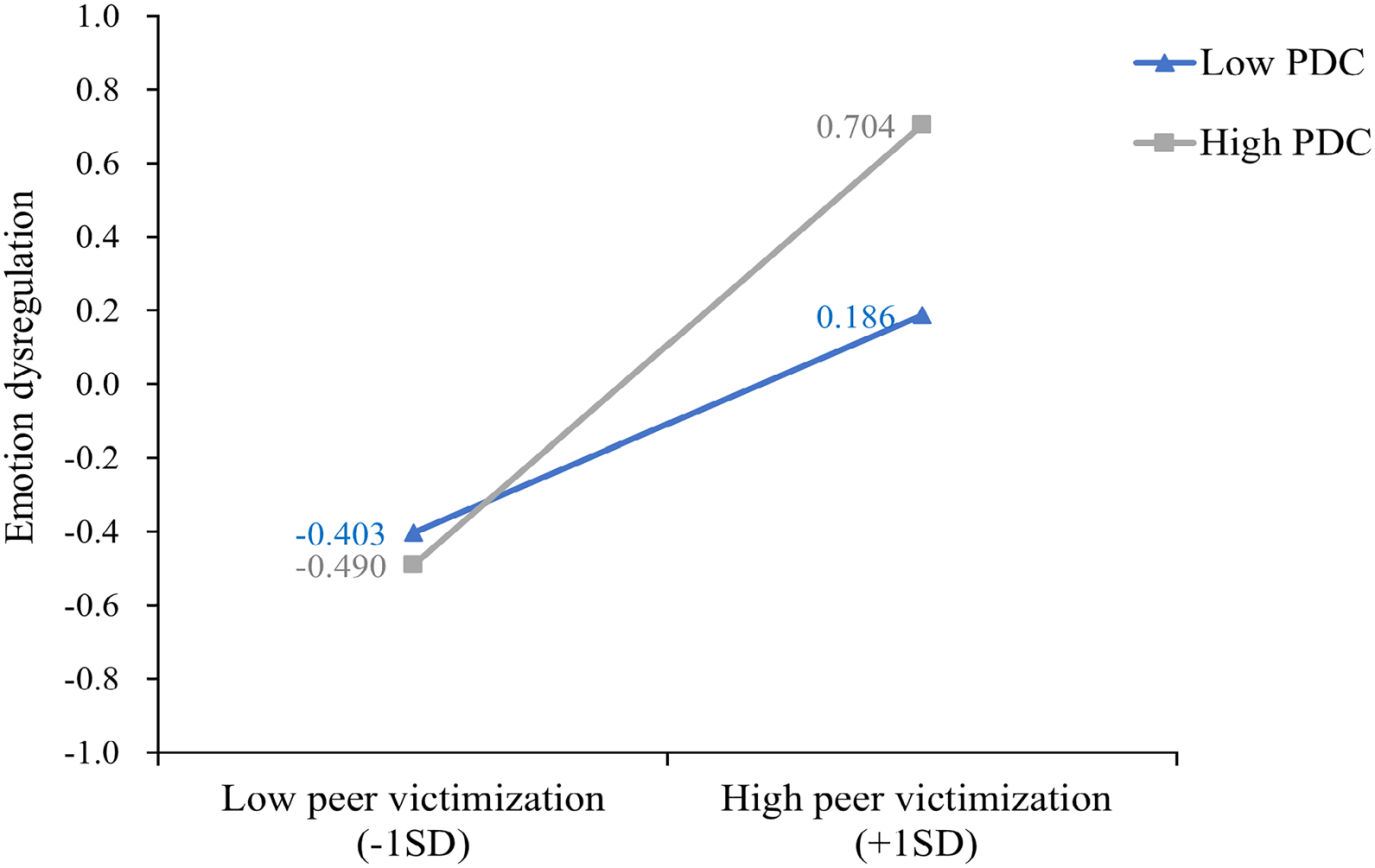
Interaction of peer victimization and polygenic dopamine composite on adolescent girls’ emotion dysregulation

**Fig. 4:**
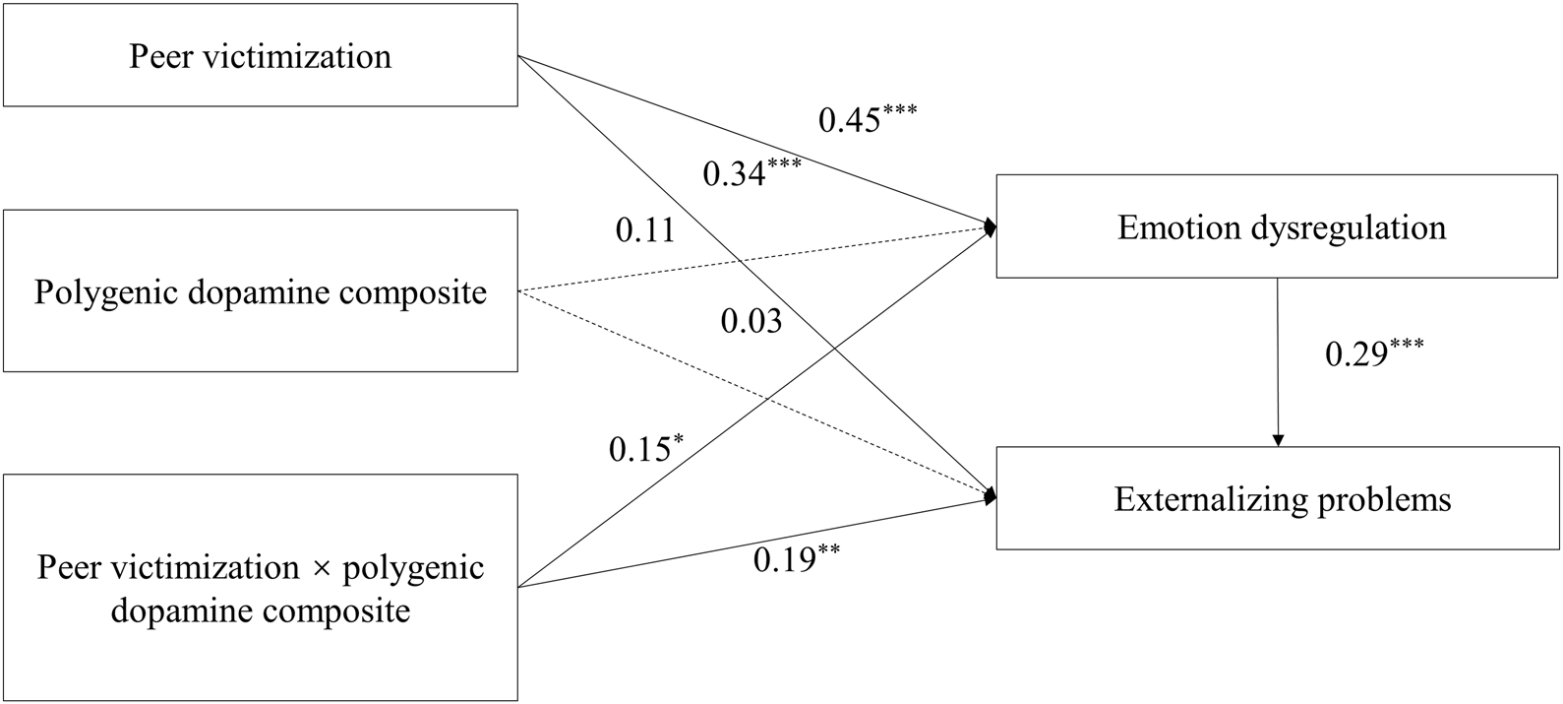
The mediated moderation model among adolescent girls. The dotted lines represent paths that are not significant; ^*^p < 0.05, ^**^p < 0.01, ^***^p < 0.001

In addition, mediated moderation hypotheses would be further supported if the moderating roles of polygenic dopamine composite were comparable in form for the proposed mediator (i.e., emotion dysregulation) and outcome (i.e., externalizing symptoms). Thus, to examine the similarities of the interactions, the same procedures employed for characterizing the interaction for the prediction of externalizing symptoms were used. Figure 3 presents the predicted emotion dysregulation as a function of peer victimization and polygenic dopamine composite levels. A simple slope test further shows that the relationship between peer victimization and girls’ emotion dysregulation was more significantly positive when levels of polygenic dopamine composite were high (*b*_*simple slope*_ = 0.60, *p* < 0.001) relative to the slope when levels of polygenic dopamine composite were low (*b*_*simple slope*_ = 0.29, *p* < 0.01), echoing the externalizing problems findings. The path parameters for the mediated moderation model for girls are shown in Fig. 4.

### Post hoc analyses for the specific genetic sources of moderation

Because the moderating effect of our polygenic composite may reflect the potential operation of one dopamine gene, additional analyses were conducted to examine whether the association between peer victimization and adolescent externalizing problems was moderated by variation in the DRD2 (two, one, or zero copies Del) and COMT (two, one, or zero copies Val) genotypes. The results can be found in the Additional file 1.

## Discussion

Peer victimization has been identified as a strong predictor of adolescent problem behavior, yet not all adolescents respond to this adverse experience in the same way. The goal of this study was to explore the moderating role of a polygenic dopamine composite consisting of COMT and DRD2 in the relationship between peer victimization and externalizing problems in Chinese adolescents, as mediated by emotion dysregulation, and the potential sex differences. Consistent with previous studies that have identified hypodopaminergic genes as a plasticity factor in negative environments [23, 24], our results showed that adolescents with high polygenic dopamine composite levels were more susceptible to peer victimization. Moreover, emotion dysregulation constituted a mechanism mediating the effect of the interaction of polygenic dopamine genes and peer victimization on externalizing problems. However, the mediated moderating effects were only found in girls. In accordance with the hypotheses, females with hypodopaminergic genes who experienced peer victimization showed greater increases in emotion dysregulation, leading to more externalizing problems. Post hoc analyses revealed that adolescent girls’ genetic susceptibility to peer victimization was attributable to both the DRD2 and the COMT genes. This work adds to prior findings regarding the role of genetic factors in adolescents’ vulnerability to peer victimization, suggesting that hypodopaminergic genes confer risk for girls’ externalizing problems via high levels of emotion dysregulation, while this is not the case for boys.

A large body of literature focuses on the links between peer victimization and victims’ internalizing problems (see the review by [9]). The current finding that peer victimization is associated with adolescents’ externalizing problems, which is consistent with previous research [4], contributes to our understanding of externalizing outcomes of peer victimization. These findings suggest that appropriate prevention and intervention efforts should be made to reduce peer victimization and to lessen victimized youth’s behavioral health problems. No main effects of hypodopaminergic genetic composite levels on externalizing problems were found in either boys or girls. It is possible that, compared to environmental factors, genetic factors have a weaker effect on community adolescents’ externalizing problems [26]. However, the results of the moderation analyses indicate a significant interaction between the hypodopaminergic composite and peer victimization with adolescent externalizing problems. Specifically, the relation between peer victimization and externalizing problems was significantly stronger among girls exhibiting higher scores for the hypodopaminergic genetic composite. In accordance with previous research (e.g., [13]), our results indicate that individuals carrying genetic alleles linked to low dopamine activity were more sensitive to adverse environments. As the biosocial developmental model suggests, low dopamine activity in mesocorticolimbic projection leads to deficits in the adolescent emotional-motivational system, increasing individuals’ sensitivity to adverse environments, which in turn confers a risk for problem behaviors [7]. Given that the polygenic composite examined in this study involved dopaminergic genes in both the mesolimbic and mesocortical pathways, it is possible that hypodopaminergic genetic dispositions amplify girls’ sensitivity to negative peer experiences, leading to more externalizing problems in contexts of increased victimization.

To understand why hypodopaminergic genes moderate girls’ responses to peer victimization, our second aim was to examine whether emotional mechanisms account for the moderating effect of polygenic dopamine composite levels. Consistent with the developing transdiagnostic models of psychopathology [29], our results imply that emotion dysregulation can be considered a potential mechanism through which dopaminergic genes interact with adverse peer experiences to predict adolescents’ externalizing problems. Extant research has identified peer victimization as an important contributing factor to dysfunctional emotional regulation [33]. Exposure to negative peer behaviors (e.g., physical attacks and relational aggression) can elicit sustained negative emotions that might deleteriously affect adolescents’ emotion regulation capabilities [50]. In turn, the inability to regulate emotions in an adaptive way contributes to more externalizing problems [51, 52].

In accordance with our hypothesis, the results indicated that the polygenic dopamine composite interacted with peer victimization to increase emotion dysregulation among girls. The pattern of the moderation of emotion dysregulation was similar to that for externalizing problems; that is, girls with high hypodopaminergic gene composite levels evidenced more emotion dysregulation than those low in dopamine composite levels when experiencing increased peer victimization. It has been hypothesized that the cortical-limbic circuit (e.g., ventral striatum, ventral tegmental area, and PFC) plays a critical role in emotion regulation [53]. The function of the mesocorticolimbic dopamine system could directly modulate reactivity to emotional environmental cues [11]. In support of this, research has shown that genetic alleles encoding low dopamine tone are associated with greater susceptibility to emotion-related impulsivity in youth with childhood adversity [54] and to emotional insecurity in adverse parenting contexts [24]. Therefore, reduced dopaminergic tone in mesolimbic and mesocortical systems may potentiate difficulties regulating emotions in contexts of peer victimization, which, in turn, could increase externalizing problems.

To delineate the specific moderating roles of the individual genes, our post hoc analyses further showed that the susceptibility effects were primarily attribute to the DRD2 gene, and the COMT gene failed to moderate the effect of peer victimization on emotional dysregulation. One possibility for the robust moderating effects of DRD2 is that the DRD2 gene is broadly expressed in multiple dopaminergic pathways [16], while the COMT gene mainly influences dopamine levels in mesolimbic circuits. Therefore, the DRD2 alleles associated with lower dopaminergic tone may be essential to neuron processes that underlie a broader range of phenotypes reflecting greater sensitivity to environment [11, 22, 55, 56]. The results furthermore supported that relying on single candidate genes could lead to erratic results, while polygenic approach could examine stronger and more consistent genetic moderation effects.

Interestingly, an interaction between dopaminergic genes and peer victimization for externalizing problems of boys was not found, which appears to stand in contrast to other research finding moderating effects of dopaminergic genes on boys’ susceptibility to the environment (e.g., [26, 57]). It is possible that the effects of oestrogen on dopamine activity contribute to sex differences in the G × E interaction results. It has been suggested that estrogen could affect the expression of dopaminergic genes (e.g., DRD2) in the mesocortical pathway [58]. Given the lower expression of dopaminergic genes in boys, dopaminergic genes may not play an adequate moderating role in the links between environmental stress and boys’ emotional and behavioral problems. However, the null finding of boys could also be a result of including only two candidate genes when considering a complex trait such as externalizing problems. Therefore, the results should be interpreted with caution regarding their generalizability. Future research is needed to get robust conclusions on these G × E interaction.

The strengths of this study include its use of a polygenic composite, examination of sex differences in the G × E interactive effects in an adolescent sample, and extension of our understanding of G × E relationships, which have mostly been investigated in family systems, by examining the role of peer-related stressors and their association with genes. Despite these strengths, several limitations of this study must be considered. First, the results are based on cross-sectional data that make limited claims about causal relationships. To demonstrate the true mediating effect of emotion dysregulation on the association between peer victimization and externalizing problems, future studies may benefit from applying longitudinal designs. Second, the current study used a sample of Chinese adolescents. Previous research has shown that the G × E interaction effects vary by ethnicity (e.g., [59, 60]), and the minor allele frequencies for COMT variants differ across different populations. We examined the allele frequency and genotype distribution of the DRD2 and COMT polymorphisms and found that the current Chinese Han sample had less Met alleles of COMT gene Val158Met polymorphism (26.0% vs. 50.8%, *χ*^2^ (1) = 8.05, *p* < 0.01) than European samples [61]. There was no significant gene frequency difference for the DRD2 gene -141C Ins/Del polymorphism (*χ*^2^ (1) = 0.35, *p* = 0.55). Thus, future research is needed to test the replicability of our findings in other ethnicities. Third, the current research used a composited genetic score based on two candidate genes. Although cumulative gene scores approach in G × E research is more comprehensive than a single candidate gene approach, future research is encouraged to apply state-of-the-art G × E techniques (e.g., genome-wide approach) to advance the field [62]. Fourth, the G × E interaction effect was only found in girls in this study, but this does not mean that boys’ responses to peer victimization are not affected by genetic factors. In contrast, studies have found that other genetic factors interact with peer victimization to predict depressive symptoms in male adolescents (e.g., [26]). Future research should further explore the combined effects of other genes and the environment on externalizing problems in adolescent boys.

## Conclusion

Extending previous G × E findings for single candidate genes, this study demonstrates that two dopamine-related genes (i.e., COMT and DRD2) collectively serve as susceptible factors that modulate the relationship between peer victimization and externalizing problems. Additionally, mediated moderation analyses suggest that the increase in emotion dysregulation accounts for the sensitivity to peer victimization of girls who carry hypodopaminergic genetic alleles. Based on a process-oriented model, these findings may advance and refine targets of prevention and intervention programs for reducing adjustment problems of adolescents with exposure to peer victimization. Consistent with previous work, these findings highlight the importance of intervention programs against peer victimization. In addition, targeting adolescents’ difficulties in emotion regulation may be a key area for intervention in reducing adolescents’ externalizing problems. Meanwhile, it is also important to consider individual differences such as those relating to genetic susceptibility.

## Supplementary Information

**Additional file 1: Table S1.** Testing the moderation model with DRD2 and COMT in adolescent girls. **Table S2.** Testing the mediated moderation model with DRD2 in adolescent girls. **Table S3.** Testing the mediated moderation model with COMT in adolescent girls.** Fig. S1.** Interaction of peer victimization and DRD2 on adolescent girls’ externalizing problems. **Fig. S2.** Interaction of peer victimization and COMT on adolescent girls’ externalizing problems.** Fig. S3.** Interaction of peer victimization and DRD2 on adolescent girls’ emotion dysregulation.

## Data Availability

The datasets generated during and/or analysed during the current study are available from the corresponding author on reasonable request. Please contact liuxia@bnu.edu.cn.
