# Supplementary material for: Examining how and why polygenic dopamine composite levels moderate adolescents’ vulnerability to peer victimization
Source: Child Adolesc Psychiatry Ment Health. 2022 Nov 17;16:84. doi: 10.1186/s13034-022-00521-7 (PMC9670640; doi:10.1186/s13034-022-00521-7)
Supplement: Supplementary file 1 — Additional file 1: Table S1. Testing the moderation model with DRD2 and COMT in adolescent girls. Table S2. Testing the mediated moderation model with DRD2 in adolescent girls. Table S3. Testing the mediated moderation model with COMT in adolescent girls. Fig. S1. Interaction of peer victimization and DRD2 on adolescent girls’ externalizing problems. Fig. S2. Interaction of peer victimization and COMT on adolescent girls’ externalizing problems. Fig. S3. Interaction of peer victimization and DRD2 on adolescent girls’ emotion dysregulation. [file 13034_2022_521_MOESM1_ESM.docx]

**Examining how and why polygenic dopamine composite levels moderate adolescents’ vulnerability to peer victimization**

**Post hoc analyses for the specific genetic sources of moderation**

Post hoc analyses were conducted to examine whether the association between peer victimization and adolescent externalizing problems was moderated by variation in the DRD2 (two, one, or zero copies Del) and COMT (two, one, or zero copies Val) genotypes. Analytic methods were identical to the analyses in Table 4 and Table 5 except that the polygenic dopamine composite was replaced by each of the specific genotypes. The results of the moderation models in Table S1 revealed that the PV × Genotype interaction significantly predict girls’ externalizing problems for the DRD2 (*β* = 0.19, *p* < 0.01) and COMT (*β* = 0.14, *p* < 0.05). Consistent with moderating effects for the polygenic dopamine composite, simple slope analyses indicated that peer victimization was more strongly associated with externalizing problems for girls with high score of DRD2 (*b_simple slope_* = 0.33, *p* < 0.001) than for girls with low score of DRD2 (*b_simple slope_* = 0.63, *p* < 0.001) (see Fig. S1), and more strongly for girls with high score of COMT (*b_simple slope_* = 0.33, *p* < 0.001) than low score of COMT (*b_simple slope_* = 0.59, *p* < 0.001) (see Fig. S2).

Further moderated mediation model analyses were conducted to test whether emotional dysregulation accounted for why peer victimization was a stronger predictor of externalizing problems for girls carrying more hypodopaminergic alleles. The results indicated that the interaction between peer victimization and the DRD2 genotype significantly predicted adolescent girls’ emotional dysregulation (*β* = 0.20, *p* < 0.01) (see Table S2), while the interaction between peer victimization and COMT did not (*β* = 0.03, *p* = 0.59) (see Table S3). Simple slope analyses for DRD2 revealed that peer victimization was more strongly associated with emotional dysregulation for girls with high score of DRD2 (*b_simple slope_* = 0.31, *p* < 0.001) than for girls with low score of DRD2 (*b_simple slope_* = 0.64, *p* < 0.001) (see Fig. S3), which is consistent with the results of DRD2 as a moderator of externalizing problems.

**Table S1** Testing the moderation model with DRD2 and COMT in adolescent girls

| Variable | Equation 1 (Externalizing Problems) | | | | | |
| --- | --- | --- | --- | --- | --- | --- |
|  | DRD2 | | | COMT | | |
|  | *β* | *SE* | *95% CI* | *β* | *SE* | *95% CI* |
| PV | 0.45^***^ | 0.06 | [0.32, 0.57] | 0.47^***^ | 0.06 | [0.34, 0.59] |
| Genotype | -0.004 | 0.06 | [-0.13, 0.12] | 0.09 | 0.06 | [-0.03, 0.22] |
| PV × Genotype | 0.19^**^ | 0.07 | [0.06, 0.32] | 0.14^*^ | 0.06 | [0.02, 0.27] |
| SES | 0.03 | 0.06 | [-0.09, 0.16] | 0.05 | 0.06 | [-0.07, 0.18] |
| *R^2^* | 0.23 |  |  | 0.23 |  |  |
| *F* | 14.60^***^ |  |  | 14.48^***^ |  |  |

*Note.* PV represents peer victimization; ^*^*p* < 0.05, ^**^*p* < 0.01, ^***^*p* < 0.001

**
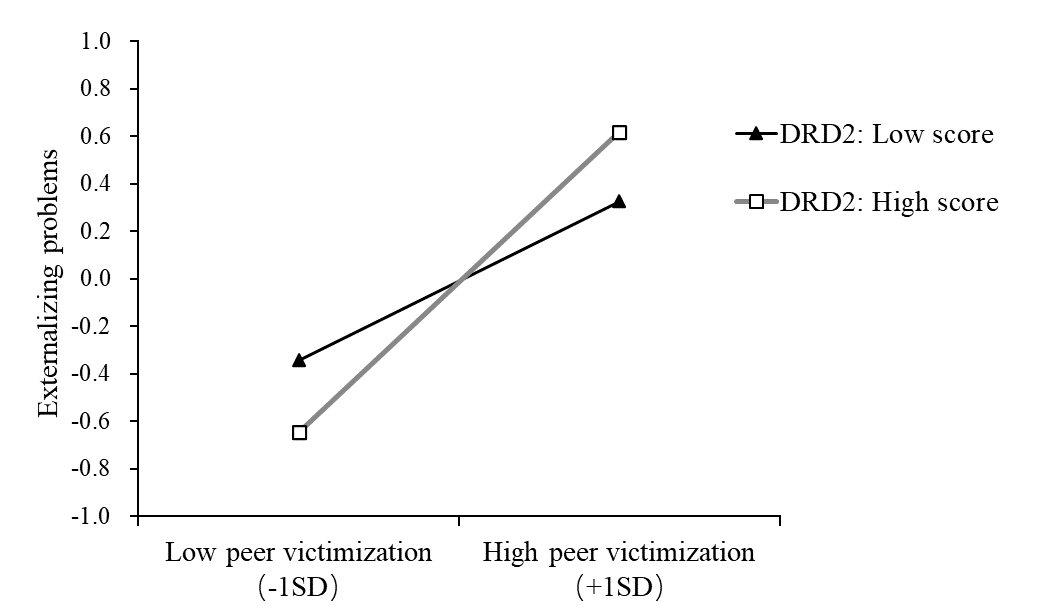
**

**Fig. S1** Interaction of peer victimization and DRD2 on adolescent girls’ externalizing problems

**
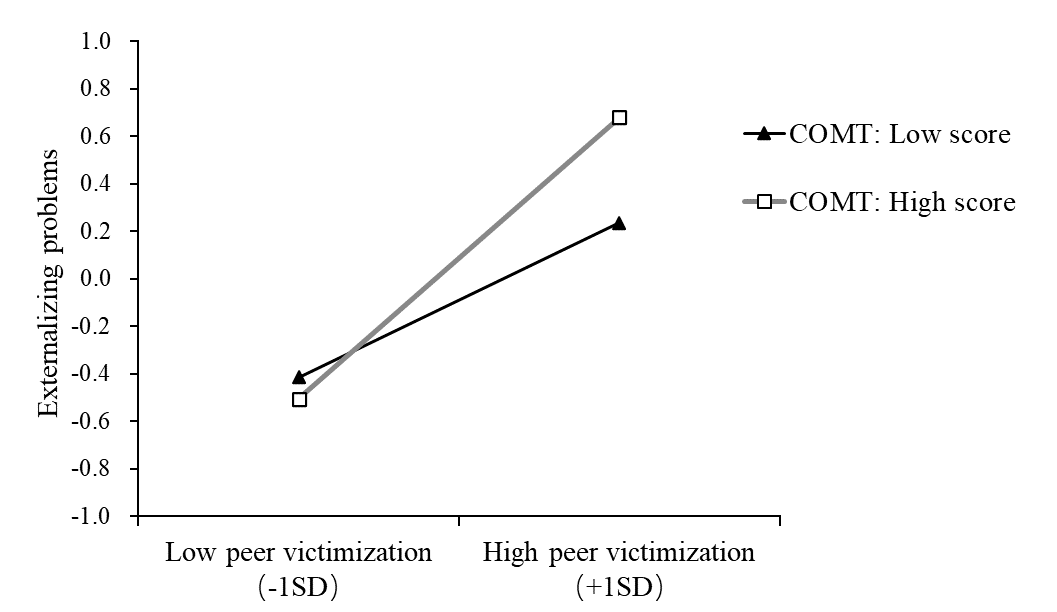
**

**Fig. S2** Interaction of peer victimization and COMT on adolescent girls’ externalizing problems

**Table S2** Testing the mediated moderation model with DRD2 in adolescent girls

| Variable | Equation 2 (Emotional dysregulation) | | | Equation 3 (Externalizing problems) | | |
| --- | --- | --- | --- | --- | --- | --- |
|  | *β* | *SE* | *95% CI* | *β* | *SE* | *95% CI* |
| PV | 0.43^***^ | 0.06 | [0.31, 0.56] | 0.31^***^ | 0.07 | [0.18, 0.45] |
| DRD2 | -0.06 | 0.06 | [-0.19, 0.06] | 0.01 | 0.06 | [-0.11, 0.13] |
| PV × DRD2 | 0.20^**^ | 0.07 | [0.07, 0.34] | 0.13 | 0.07 | [-0.003, 0.26] |
| ED |  |  |  | 0.30^***^ | 0.07 | [0.16, 0.44] |
| SES | 0.001 | 0.06 | [-0.12, 0.13] | 0.03 | 0.05 | [-0.09, 0.15] |
| *R^2^* | 0.23 |  |  | 0.30 |  |  |
| *F* | 14.24^***^ |  |  | 16.57^***^ |  |  |

*Note.* PV and ED represent peer victimization and emotion dysregulation respectively; ^*^*p* < 0.05, ^**^*p* < 0.01, ^***^*p* < 0.001.

**
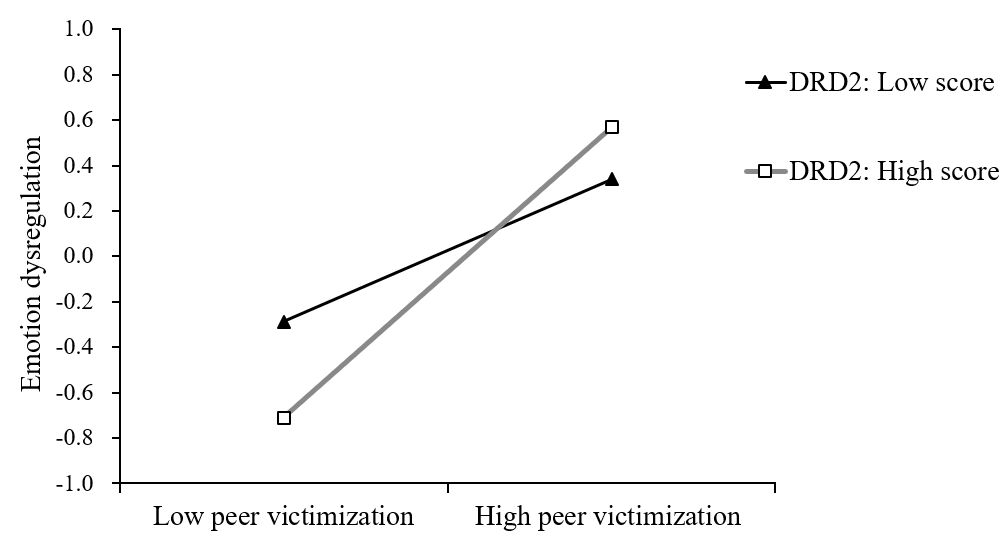
**

**Fig. S3** Interaction of peer victimization and DRD2 on adolescent girls’ emotion dysregulation

**Table S3** Testing the mediated moderation model with COMT in adolescent girls

| Variable | Equation 2 (Emotional dysregulation) | | | Equation 3 (Externalizing problems) | | |
| --- | --- | --- | --- | --- | --- | --- |
|  | *β* | *SE* | *95% CI* | *β* | *SE* | *95% CI* |
| PV | 0.45^***^ | 0.06 | [0.32, 0.57] | 0.33^***^ | 0.07 | [0.19, 0.46] |
| COMT | 0.20^**^ | 0.06 | [0.07, 0.32] | 0.03 | 0.06 | [-0.09, 0.15] |
| PV × COMT | 0.03 | 0.06 | [-0.09, 0.16] | 0.13^*^ | 0.06 | [0.01, 0.25] |
| ED |  |  |  | 0.31^***^ | 0.07 | [0.18, 0.45] |
| SES | 0.01 | 0.06 | [-0.11, 0.14] | 0.03 | 0.05 | [-0.08, 0.17] |
| *R^2^* | 0.23 |  |  | 0.31 |  |  |
| *F* | 14.24^***^ |  |  | 16.92^***^ |  |  |

*Note.* PV and ED represent peer victimization and emotion dysregulation respectively; ^*^*p* < 0.05, ^**^*p* < 0.01, ^***^*p* < 0.001.
